# New Criteria for Functions to Be in a Class of *p*-Valent Alpha Convex Functions

**DOI:** 10.1155/2013/280191

**Published:** 2013-09-26

**Authors:** Muhammad Arif, Muhammad Ayaz, Mohamed Kamal Aouf

**Affiliations:** ^1^Department of Mathematics, Abdul Wali Khan University, Mardan 23200, Pakistan; ^2^Faculty of Science, Mansoura University, Mansoura 35516, Egypt

## Abstract

We obtain certain simple sufficiency criteria for a class of *p*-valent alpha convex functions. Many known results appear as special consequences of our work. Some applications of our work to the generalized integral operator are also given.

## 1. Introduction

Let *A*_*p*_(*n*) denote the class of functions *f*(*z*) of the form

(1) $$\begin{array}{c} f\left(z\right)=z^{p}+\sum_{k=p+n}^{\infty }a_{k}z^{k}\;\left(p\in \mathbb{N}=\left\{1,2,\ldots \right\}\right), \end{array}$$

which are analytic and multivalent in the open unit disk *𝕌* = {*z* ∈ *ℂ* : |*z*| < 1}. We note that *A*_1_(*n*) = *A*(*n*) and *A*_1_(1) = *A*. Also let *S*_p_*(*n*, *β*), *C*_*p*_(*n*, *β*) and *M*_*p*_(*α*, *n*, *β*), (0 ≤ *α* ≤ 1, 0 ≤ *β* < *p*,  *n*,*p* ∈ *ℕ*), denote the subclasses of *A*_*p*_(*n*) consisting of all functions *f*(*z*), of the form (1), which are defined, respectively, by

(2) Rezf′(z)f(z)>β, (z∈𝕌),1+Rezf′′(z)f′(z)>β, (z∈𝕌),Re{(1−α)zf′(z)f(z)+αzf′′(z)f′(z)}>β−α, (z∈𝕌).

For *p* = 1 and *n* = 1, the above three classes reduce to the well-known classes of starlike, convex, and alpha convex functions of order *β*, respectively. We also note that *M*_1_(0, *n*, 0) ≡ S*(*n*, 0), *M*_1_(1, *n*, 0) ≡ *C*(*n*, 0).

Sufficient condition was studied by various authors for different subclasses of analytic and multivalent functions; for some of the related work, see [1–9]. The object of the present paper is to obtain sufficient conditions for the class *M*_*p*_(*α*, *n*, *β*) of multivalent alpha convex functions of order *β*. We also consider some special cases of our results which lead to various interesting corollaries, and relevances of some of these with other known results are also mentioned.

We will assume throughout our discussion, unless otherwise stated, that *n* ∈ *ℕ*, 0 ≤ *α* ≤ 1, 0 ≤ *β* < *p*, and *p* ∈ *ℕ*.

To obtain our main results, we need the following lemmas due to Mocanu [10].

**Lemma 1**

If *q*(*z*) ∈ *A*(*n*) satisfies the condition

(3) $$\begin{array}{c} \left|q^{\prime }\left(z\right)-1\right|<\frac{n+1}{\sqrt{{\left(n+1\right)}^{2}+1}}\;\left(z\in 𝕌\right), \end{array}$$

then

(4) $$\begin{array}{c} q\left(z\right)\in S^{*}\left(n,0\right). \end{array}$$

**Lemma 2**

If *q*(z) ∈ *A*(*n*) satisfies the condition

(5) $$\begin{array}{c} \left|\arg q^{\prime }\left(z\right)\right|<\frac{\pi }{2}\delta_{n}\;\left(z\in 𝕌\right), \end{array}$$

where *δ*_*n*_ is the unique root of the equation

(6) $$\begin{array}{c} 2\tan^{-1}\left[n\left(1-\delta_{n}\right)\right]+\pi \left(1-2\delta_{n}\right)=0, \end{array}$$

then

(7) $$\begin{array}{c} q\left(z\right)\in S^{*}\left(n,0\right). \end{array}$$

## 2. Sufficient Conditions for the Class *M*_*p*_(*α*,  *n*, *β*)

**Theorem 3**

If *f*(*z*) ∈ *A*_*p*_(*n*) satisfies

(8) |(f(z)zp(zf′(z)pf(z))α)1/(p−β) ×{(1−α)zf′(z)f(z)+αzf′′(z)f′(z)+α−β}−p+β|  <n+1(n+1)2+1(p−β) (z∈𝕌),

then *f*(*z*) ∈ *M*_*p*_(*α*, *n*, *β*).

**Proof**

Let us set a function *q*(*z*) by

(9) q(z)=z(f(z)zp(zf′(z)pf(z))α)1/(p−β)=z+α(p+n)ap+np(p−β)zn+1+⋯,

for *f*(*z*) ∈ *A*_*p*_(*n*). Then clearly (9) shows that *q*(*z*) ∈ *A*(*n*).

Differentiating (9) logarithmically, we have

(10) q′(z) =q(z)[1z+1p−β{(1−α)f′(z)f(z)+αf′′(z)f′(z)−p−αz}],

which gives

(11) |q′(z)−1|=|(f(z)zp(zf′(z)pf(z))α)1/(p−β)1p−β×{(1−α)zf′(z)f(z)+αzf′′(z)f′(z)+α−β}−1|.

Thus using (8), we have

(12) $$\begin{array}{c} \left|q^{\prime }\left(z\right)-1\right|\leq \frac{n+1}{\sqrt{{\left(n+1\right)}^{2}+1}},\;\left(z\in 𝕌\right). \end{array}$$

Hence, using Lemma 1, we have *q*(*z*) ∈ S*(*n*, 0).

From (10), we can write

(13) $$\begin{array}{c} \frac{zq^{\prime }\left(z\right)}{q\left(z\right)}=1+\frac{1}{p-\beta }\left\{\left(1-\alpha \right)\frac{zf^{\prime }\left(z\right)}{f\left(z\right)}+\alpha \frac{zf^{\prime \prime }\left(z\right)}{f^{\prime }\left(z\right)}+\alpha -p\right\}. \end{array}$$

Since *q*(*z*) ∈ S*(*n*, 0), it implies that *Re*(*zq*′(*z*)/*q*(*z*)) > 0. Therefore, we get

(14) 1p−βRe{(1−α)zf′(z)f(z)+α(1+zf′′(z)f′(z))−β} =Rezq′(z)q(z)>0,

or

(15) 1p−βRe{(1−α)zf′(z)f(z)+α(1+zf′′(z)f′(z))−β}>0,

and this implies that *f*(*z*) ∈ *M*_*p*_(*α*, *n*, *β*).

By taking *α* = 0 and *α* = 1 in Theorem 3, we obtain Corollaries 4 and 5 proved by Goyal et al. [6].

**Corollary 4**

If *f*(*z*) ∈ *A*_*p*_(*n*) satisfies

(16) |(f(z)z)1/(p−β){z(1−β)/(p−β)f′(z)f(z)−βz(1−p)/(p−β)  }−p+β| <n+1(n+1)2+1(p−β) (z∈𝕌),

for 0 ≤ *β* < *p*, then *f*(*z*) ∈ *S*_p_*(*n*, *β*).

**Corollary 5**

If *f*(*z*) ∈ *A*_*p*_(*n*) satisfies

(17) |{(f′(z))β+1−ppzp−1}1/(p−β){zf′′(z)+(1−β)f′(z)}−p+β| <n+1(n+1)2+1(p−β) (z∈𝕌),

for 0 ≤ *β* < *p*, then *f*(*z*) ∈ *C*_*p*_(*n*, *β*).

Further if we take *n* = 1 and *p* = 1 in Corollaries 4 and 5, we get the following result proved by Uyank et al. [9].

**Corollary 6**

If *f*(*z*) ∈ *A* satisfies

(18) |(f(z)z)1/(1−β){zf′(z)f(z)−β}−1+β| <25(1−β) (z∈𝕌),

for 0 ≤ *β* < 1, then *f*(*z*) ∈ S*(*β*).

**Corollary 7**

If *f*(*z*) ∈ *A* satisfies

(19) |(f′(z))β/(1−β){f′(z)+11−βzf′′(z)}−1| <25 (z∈𝕌),

for 0 ≤ *β* < 1, then *f*(*z*) ∈ *C*(*β*).

**Remark 8**

If we put *β* = 0 and *p* = 1 in Corollaries 4 and 5, we get the result proved by Mocanu [7] and Nunokawa et al. [8], respectively.

**Theorem 9**

If *f*(*z*) ∈ *A*_*p*_(*n*) satisfies

(20) |arg{f(z)zp(zf′(z)pf(z))α} +(p−β)arg{(1−α)zf′(z)f(z)+αzf′′(z)f′(z)+α−β}|  <π2δn(p−β) (z∈𝕌),

where *δ*_*n*_ is the unique root of (6), then *f*(*z*) ∈ *M*_*p*_(*α*, *n*, *β*).

**Proof**

Let us set

(21) q(z)=z(f(z)zp(zf′(z)pf(z))α)1/(p−β)=z+α(p+n)ap+np(p−β)zn+1+⋯,

for *f*(*z*) ∈ *A*_*p*_(*n*). Then clearly (9) shows that *q*(*z*) ∈ *A*(*n*).

Differentiating (9), we have

(22) q′(z) =q(z)z{1(p−β)       ×{(1−α)zf′(z)f(z)+αzf′′(z)f′(z)+α−p}+1},

which gives

(23) |argq′(z)| =|argq(z)z   +arg{(1−α)zf′(z)f(z)+αzf′′(z)f′(z)+α−β}|.

Thus using (8), we have

(24) $$\begin{array}{c} \left|\arg q^{\prime }\left(z\right)\right|\leq \frac{\pi }{2}\delta_{n}\;\left(z\in 𝕌\right), \end{array}$$

where *δ*_*n*_ is the root of (6). Hence, using Lemma 2, we have *q*(*z*) ∈ S*(*n*, 0). Now by the same arguments as in the proof of Theorem 3, we obtain the required result.

Making *α* = 0 in Theorem 9, we have the following.

**Corollary 10**

If *f*(*z*) ∈ *A*_*p*_(*n*) satisfies

(25) |arg(f(z)zp)+(p−β)arg{zf′(z)f(z)−β}| <π2δn(p−β) (z∈𝕌),

then *f*(*z*) ∈ *S*_p_*(*n*, *β*).

Further if we take *n* = 1 = *p* in Corollary 10, we get the following result proved by Uyank et al. [9].

**Corollary 11**

If *f*(*z*) ∈ *A* satisfies

(26) |arg(f(z)z)+(1−β)arg{zf′(z)f(z)−β}| <π2δ1(1−β) (z∈𝕌),

where *δ*_1_ is the unique root of the equation

(27) $$\begin{array}{c} 2\tan^{-1}\left[\left(1-\delta_{1}\right)\right]+\pi \left(1-2\delta_{1}\right)=0, \end{array}$$

then *f*(*z*) belongs to the class of starlike functions of order *β*.

Taking *α* = 1 in Theorem 9, we have the following.

**Corollary 12**

If *f*(*z*) ∈ *A*_*p*_(*n*) satisfies

(28) |arg(f′(z)pzp−1)+(p−β)arg{zf′′(z)f′(z)+1−β}| <π2δn(p−β), (z∈𝕌),

then *f*(*z*) ∈ *C*_*p*_(*n*, *β*).

**Remark 13**

If we take *n* = 1 and *p* = 1 in Corollary 12, we get the result proved in [9], and further for *β* = 0, we get the result proved by Mocanu [10].

## 3. Generalized Integral Operator

For *f*(*z*) ∈ *A*_*p*_(*n*), we consider

(29) $$\begin{array}{c} G\left(z\right)={\left\{\delta \int_{0}^{z}t^{\delta -1}{\left(\frac{f\left(t\right)}{t^{p}}\right)}^{\gamma }dt\right\}}^{1/\delta }=z+\frac{\gamma a_{p+n}}{\left(n+\delta \right)}z^{n+1}+\cdots . \end{array}$$

Clearly *G*(*z*) ∈ *A*(*n*), and when *p* = 1, *γ* = 1, and *δ* = 1, then (29) reduces to the well-known Alexander integral operator [11].

**Theorem 14**

If *γ* ≥ *δ*/*p*, *δ* > 0, and *f*(*z*) ∈ *A*_*p*_(*n*) satisfy

(30) $$\begin{array}{c} \left|{\left(\frac{f\left(z\right)}{z^{p}}\right)}^{\gamma /\delta }\left\{1+\frac{\gamma }{\delta }\left(\frac{zf^{\prime }\left(z\right)}{f\left(z\right)}-p\right)\right\}-1\right|\leq \frac{\left(n+1\right)}{\sqrt{{\left(n+1\right)}^{2}+1}}, \end{array}$$

then *f*(*z*) ∈ *S*_p_*(*n*, 0).

**Proof**

From (29), we get

(31) $$\begin{array}{c} G^{\delta -1}\left(z\right)G^{\prime }\left(z\right)=z^{\delta -1}{\left(\frac{f\left(z\right)}{z^{p}}\right)}^{\gamma }. \end{array}$$

Differentiating (31), logarithmically, we get

(32) $$\begin{array}{c} \left(\delta -1\right)\frac{G^{\prime }\left(z\right)}{G\left(z\right)}+\frac{G^{\prime \prime }\left(z\right)}{G^{\prime }\left(z\right)}=\left(\delta -1\right)\frac{1}{z}+\gamma \left(\frac{f^{\prime }\left(z\right)}{f\left(z\right)}-\frac{p}{z}\right). \end{array}$$

Then by simple computation, we have

(33) |G(z)z(zG′(z)G(z))1/δ ×{(1−1δ)zG′(z)G(z)+1δ(zG′′(z)G′(z)+1)}−1| =|(f(z)zp)γ/δ{1+γδ(zf′(z)f(z)−p)}−1| ≤(n+1)(n+1)2+1,

where we have used (30). By using Theorem 3 with *p* = 1, *β* = 0, and *α* = 1/*δ*, we have  *G*(*z*) ∈ *M*_1_(1/*δ*, *n*, 0).

From (32), we can write

(34) Re{(1−1δ)zG′(z)G(z)+1δ(zG′′(z)G′(z)+1)} =γδRezf′(z)f(z)−pγδ+1,

or

(35) Rezf′(z)f(z)>(p−δγ) (since  G(z)∈M1(1δ,n,0)),

which shows that *f*(*z*) ∈ *S*_p_*(*n*, 0), where *γ* ≥ *δ*/*p*.

**Theorem 15**

If *γ* ≥ *δ*/*p*, *δ* > 0, and *f*(*z*) ∈ *A*_*p*_(*n*) satisfy

(36) $$\begin{array}{c} \left|\arg{\left(\frac{f\left(z\right)}{z^{p}}\right)}^{\gamma /\delta }+\arg\left\{\frac{\gamma }{\delta }\left(\frac{zf^{\prime }\left(z\right)}{f\left(z\right)}-p\right)+1\right\}\right|<\frac{\pi }{2}\delta_{n}, \end{array}$$

where *δ*_*n*_ is the unique root of (6), then *f*(*z*) ∈ *S*_p_*(*n*, 0).

**Proof**

The result follows on similar lines as in the last theorem using Theorem 9 with *p* = 1, *β* = 0, and *α* = 1/*δ*.
